# Nanotechnology in Fire Protection—Application and Requirements

**DOI:** 10.3390/ma14247849

**Published:** 2021-12-18

**Authors:** Anna Rabajczyk, Maria Zielecka, Tomasz Popielarczyk, Tomasz Sowa

**Affiliations:** Scientific and Research Center for Fire Protection, National Research Institute, Nadwiślańska 213, 05-420 Jozefow, Poland; mzielecka@cnbop.pl (M.Z.); tpopielarczyk@cnbop.pl (T.P.); tsowa@cnbop.pl (T.S.)

**Keywords:** nanomaterials, fire protection, direct and indirect protection, threats

## Abstract

Nanotechnology is used, to an increasing extent, in practically every aspect of the economy and society. One area where nanotechnology is constantly advancing is fire protection. Nanostructures are found in elements used in direct protection, such as in protective clothing, filters, and helmets. Solutions in the field of nanotechnology are also used in elements reducing the fire risk and increasing the fire safety, such as building materials and structures, paints, coatings, or fire safety equipment (e.g., fire detectors). However, new solutions may also pose a threat to the safety of people and the environment. As a result of operation or combustion and degradation processes, the emission of nano-substances with toxic properties may occur. Therefore, knowledge in this field is necessary, as it allows for the appropriate targeting and use of nanotechnology.

## 1. Fire Protection—Introduction

Fire protection is related to activities aimed at protecting life, health, property, and the environment against fire and its consequences. Protection measures refer both to areas related to fire prevention and the protection of people and property during an emergency. They are used both in the area of direct and indirect actions and include, inter alia, elements such as: exercises related to safety planning or education in the field of fire and safety hazards, training and testing of mitigation systems. There are three basic principles of fire protection including fire study, active fire protection, and passive fire protection. In the field of fire risk analysis, analysis of the causes of the fire, types of extinguishing techniques, and their possible applications in a given situation, as well as the tools used in actions, i.e., detection and extinguishing equipment, and their applications are covered. The study also includes the definition of rules and regulations related to the construction of buildings and the type of materials used. In the case of active protection, the analysis covers both manual and automatic fire detection systems, fire alarms and their application, firefighting, and first aid. Passive fire protection, on the other hand, refers to issues related to the design of buildings and infrastructure, the possibility of using fireproof materials, adequate fire insulation, separation walls and fire doors, and smoke-tight doors. Passive protection also includes training in fire safety, signage with safety signs, and the development of evacuation plans [1].

Rescue actions therefore refer to any action that is taken to protect life, health, property, or the environment. They also deal with activities related to the elimination of the causes of fire, natural disasters, or other local threats.

The purpose of this review is to present the most important information on the applicability of nanotechnology in fire protection, direct and indirect, based on a literature review using the following keywords: fire protection, direct protection measures, fire protection, fire protection agents and materials, flame retardant additives and explosiveness, danger to life, and health of the firefighter. The literature review was performed using the following databases: Web of Knowledge, Scopus, and Google Scholar. The research also covered Espacenet, Patentscope, and Google Patents, which resulted in the presentation of selected patents relevant to the subject. The review is divided into the following main sections, including a discussion focusing on the requirements for materials and tools used in firefighting, the impact of nano-additives in reducing the risk of fire, and the risk of nanostructures in a fire or extinguishing operation.

## 2. Legal Requirements

Appropriate fire regulations must be implemented both in living quarters or workplaces, and in public places. Each enterprise, entity, or administrative unit is obliged to ensure adequate fire protection. However, depending on the country or region, fire protection regulations are included in documents prepared by appropriate institutions. In the United States, it is the National Fire Protection Association (NFPA) [2], in Canada it is, inter alia, Canadian Center for Occupational Health and Safety (CCOHS) [3,4], which establishes regulations for workplace safety. In the European Union, however, legal provisions are contained in regulations of the European Commission. In addition, in the EU, the European Organization for Technical Approvals (EOTA) [5] is responsible for coordinating the reconciliation and issuing of European Technical Assessments and certification of products for compliance with these assessments, while The Confederation of Fire Protection Associations Europe (CFPA-Europe) [6,7] is an organization associating, among others, certification entities from European countries and associations of people involved in fire protection. In the case of Poland, fire protection issues are dealt with by the Ministry of the Interior and Administration, the National Fire Service [8] and other units established under the Fire Protection Act [9]. When developing solutions in the field of fire protection, it is important to verify what legal requirements are in force in a given area, as the scope of requirements to be met may differ (Table 1).

It should be added that the European directives are addressed mainly to manufacturers and assemblers of machines, while OSHA standards are addressed to recipients, i.e., employees operating machines. Within the European Union, the standards for tests, markings of the assessment system, rules for issuing technical assessments, and certification of construction products are harmonized, while the provisions on buildings in the field of fire protection remain differentiated. It is worth noting that depending on the product, it is necessary to adapt the construction product or the actions taken to the legal requirements in force in a given area. For example, there are a number of different guidelines for sprinkler systems including, but not limited to, PN-EN 12845 “Fixed firefighting systems–Automatic sprinkler systems–Design, installation and maintenance”, guidelines of the German insurers VdS CEA4001 “Sprinkler Systems–Planning and Installation”, American NFPA 13 “Standard for the installation of sprinkler systems”, and NFPA 20 “Standard for the installation of stationary pumps for fire protection”.

The solutions used in the field of new materials must meet the relevant guidelines, including resistance to high temperatures, resistance to chemical agents, detection precision, data transmission speed, correctness in the reflection of fire spread in buildings or in open areas, and effectiveness of neutralization of pollutants. One of the requirements for new materials is fire resistance. Materials, depending on their resistance and application, are classified differently. For example, building materials are classified as REI tt, that is [22,23]:

- Loadbearing capacity (R)—ability of the trial element of the load-bearing structure element to support the test load without exceeding certain criteria in terms of both the magnitude and the rate of deflection.
- Integrity (E)—ability of a test piece separating building structure elements to prevent the passage of flames and hot gases and to prevent the appearance of flames on an unheated surface.
- Thermal Insulation (I)—ability of a test piece separating structural elements, when exposed to fire on one side, to limit the rise in temperature of the unheated surface below specified levels.
- tt (time in minutes)— the time when all criteria are met (R, E and I) [22,23].

The European system provides in great detail the possible time classifications, which are: 15, 20, 30, 45, 60, 90, 120, 180, and 240 min. Regardless of the uniform classification system throughout Europe, national regulations and laws sometimes differ in terms of the time requirements in which all appropriate criteria for a given product must be met. Such a large amount of diversity means that both producers and building/area management units responsible for fire protection have to adapt to specific legal provisions [22,23].

In order to meet the increasingly restrictive legal requirements and standards in the field of fire protection, solutions are introduced for a wide range of materials and industrial products, sensors, and chemical compounds based on nanotechnology. It should be noted that materials or tools that are used for indirect or direct protection against the risks of explosion and fire must meet the same requirements as materials and tools without the use of nanotechnology. In the case of introducing new compounds, it is necessary to meet the REACH requirements and develop a safety data sheet for a device or material, in accordance with the requirements in these areas. Regulation of nanotechnology-based products is carried out within the framework of existing statutory bodies, in accordance with specific legal standards applicable to each type of product under the jurisdiction of a given authority [24,25]. However, it should be noted that in the event of a hazard resulting from working in conditions with the presence and the possibility of direct contact with nanosubstances, special requirements relating to safety and working conditions are developed. However, this issue will not be discussed in this study.

## 3. Nanomaterials in Personal Protection

One of the important areas of fire protection is the appropriate equipment of people participating in rescue operations, defined as the provision of personal protective equipment. In the European Union, the guidelines in this area are included in the Regulation (EU) 2016/425 of the European Parliament and of the Council of 9 March 2016 on personal protective equipment and repealing Council Directive 89/686/EEC and Directive 89/656/EEC [26], which concerns the minimum health and safety requirements for the use of personal protective equipment by employees. Personal protective equipment (PPE) is divided into the three following categories:

- Protecting against minimal risks (Category I).
- Protecting against a specific factor that does not threaten life or health and does not cause permanent damage to the health of the employee (Category II).
- Specialist clothing, the task of which is to protect against factors that may cause the most dangerous consequences for the worker, and the direct effects of which cannot be identified in a timely manner, protective clothing of complex construction to protect against the threat to life or health of the worker, including a firefighter (Category III) [26].

The scope of personal protective equipment includes devices, equipment, and clothing that are designed to ensure safety and protect against one or more hazards [27,28]. Therefore, they include protective clothing and measures to protect the lower and upper limbs, head, face, eyes, respiratory system, and hearing, as well as equipment to prevent an employee from falling from a height, and measures to isolate the entire body. All these elements must meet the criteria specified by law and standards in the field of, inter alia, resistance to temperature or chemicals, durability, possible use in open areas or indoors. External factors such as:

- Fire, hot air, heat radiation, hot water, and steam. High or low temperature.
- Chemicals, including foaming agents.
- Gases generated during combustion or leaking from the installation.
- Biological agents such as viruses, bacteria, and dangerous organisms.
- Other elements such as glass, metal, sharp objects, and electric discharges [28].

On the other hand, it is necessary to ensure that the equipment is comfortable as well as functional when undertaking activity. Such a variety of parameters that determine the safety of people taking part in various types of action require a specialized approach. For this reason, the materials of individual PPE are subject to constant modifications, including the use of nanotechnology.

### 3.1. Protective Clothing

Inherently fire-resistant fibers or chemically modified fibers with fire-resistant properties are used in protective clothing. Inherently flame retardant fibers that do not melt easily and do not ignite are compounds such as: polyamides (such as: poly(*p*-phenylene terephthalamide), imide derivatives (such as: poly(aramid-imide), polyimide, polyether imide, compounds containing azole group (such as: polybenzimidazole, polybenzoxazoles), halogenated (such as: chlorinated, fluorinated), melamine-formaldehyde, polyetheretherketones, phenolic, polyphenylene sulphide, polyacrylate, semi-carbon, glass, and ceramic [28]. It should be noted that aramid fibers, which are aromatic polyamides, are known under the trade names Kevlar™, Technora™, Twaron™ (poly(*p*-phenylene terephthalamide), PPD-T) or Nomex™, Newstar™ (poly(*m*-phenylene terephthalamide), PD-T, PPDT, or PPTA), and Conex™ (poly(*m*-phenylene isophthalamide), MPIA) [28,29,30,31,32].

These fibers tend to char or form ash when exposed to fire and therefore do not usually contribute to the overall flammability of polymer matrix composites (PMC). Aramids burn faster than carbon fibers [33]. Cellulose fibers, on the other hand, refer to natural-based fibers used in the reinforcement of PMC, typically for lower performance and biorenewable PMC composites. These types of fibers often burn with the polymer and can negatively affect the combustibility. However, in the presence of flame retardants, cellulose fibers can serve as additional sources of carbonization for fire protection in swelling formulations, which is not observed with glass fiber or carbon fiber [28,34].

The aim of these modifications is to achieve above 21% of the parameter called the limited oxygen index (LOI), indicating the minimum percentage of oxygen required for combustion [34,35]. The techniques used to produce chemically modified refractory fibers are primarily determined by the type of combustible substrate fibers in the process. Both synthetic fibers such as polyester, nylon, and acrylic, and natural fibers such as wool, cotton, and viscose are used in the modification processes. Flame retardant substances, in the case of synthetic fibers are, among others, halogens, nitrogen, silicon, and phosphorus [28,36,37]. These substances are either incorporated into the polymerization process during melt-spinning or doped into the spinning bath during solution-spun fiber preparation. Such modified fibers, in the event of exposure to high temperatures and thermal hazards, create a gas-vapor phase (a non-volatile ester compound) or a solid-state condensed phase (carbonized carbon compound), which reduces flammability [38,39]. Thereby insulation of other underlying materials takes place and helps to maintain the integrity of firefighters’ protective clothing.

Shaid et al. [39] investigated the protective properties of aerogel nonwoven fabric in firefighter’s protective clothing (FPC). The thermal lining used was a 100% woven Nomex face fabric, nonwoven fabric, and a commercial moisture barrier from Bruck Textile, Australia. The aerogel nonwoven fabric was based on silica aerogel in a flexible form, with a thermal conductivity of about 23 mW/m/K and a fabric grammage of 285 g/m^2^. The reinforcement material was the material of the company MFB (Metropolitan Fire Brigade), Australia. The results of the research indicate that aerogel nonwoven fabric can provide thermal resistance which is eight times greater than that of commercial reinforcement material and thermal material. The use of an aerogel nonwoven layer as a thermal liner resulted in a five-fold increase in heat resistance in comparison to the use of a thermal liner alone, and a three-fold increase in heat resistance than when the existing thermal liner and moisture barrier are combined. The possibility of the occurrence of burn injuries under a compressive load of 49 N on a surface heated to 200 °C was also tested. It has been found that a firefighter will experience an immediate burn within 30 s if only commercial reinforcement material is used. However, if only aerogel nonwoven fabric is used, the firefighter will have 86 s before feeling the pain, 107 s before the first-degree burn, and 2 and a half minutes before the burn. Theoretically receiving a second-degree burn in the same condition. The use of aerogel nonwoven fabric gives the firefighter more than 1 min to withdraw from the emergency. It was also found that the use of aerogel reinforcement can significantly increase the protective properties of FPC [39].

Modifications of the crystalline structure of the fibers at a specific transformation temperature is the basis of shape memory materials (SMM) which are able to change their current shape into a specific shape of the crystal structure. The shape memory material in the fireproof garment is activated by heat. The air gaps between adjacent layers of clothing widen, which provides better insulation [40]. In the case of polymers, the shape memory effect is observed when a material that adjusts to a single shape returns to the previously adopted shape at a certain temperature. Phase-change materials (PCM) are also used in firefighting protective clothing to increase thermal protection. Based on the results of the theoretical model, it was found that the inclusion of PCM in firefighting clothing would provide equivalent thermal protection with a reduced thickness of the clothing [28].

The conducted research has shown that the use of polymer nanoparticles (NPs) for the production of nanocomposites improves the mechanical, thermal, and electrical properties of fabrics [41,42,43] and reduces flammability. At the same time, it was indicated that nanoparticles should be combined with other flame retardants, as the use of only nanoparticles may reduce heat release [44,45] and improve certain anti-drip properties of thermoplastics. The heat reduction mechanism provided by the polymer nanocomposite technology ultimately reduces polymer weight loss, which in turn reduces heat (fuel) release. It should be noted in this connection that the nanoparticles may cause some processing difficulties in the production of the PMC. Nanoparticles almost always increase the viscosity of a polymer, and this increase can create serious challenges in making PMCs. In particular, the high viscosity of the resin often prevents the polymer from fully wetting the fiber, leading to voids and “dry” spots in the PMC that serve as sites for the initiation of mechanical damage. Therefore, when nanoparticles are present, which cause an increased viscosity of the resin, it can be very difficult to obtain a material with appropriate parameters [43].

The nanofibers have a large surface area per unit mass. Nonwoven mesh makes the materials breathable and thermally insulating. Coating is a common method of applying nanofibers to protective clothing [46]. While traditional textiles used in thermal protective clothing rely on a passive insulation mechanism to protect the firefighter, smart clothing can provide active protection. In the case of smart clothing, the solution is injected into the outer layer of the garment through a capillary mesh and the injection process is activated by a temperature sensor embedded in the outer layer of the cloth [47]. On exposure to fire, the injected water evaporates. High heat absorption occurs and, consequently, the temperature rise in the outer layer slows down, providing active protection against exposure to flash fire [29].

Mandal et al. [48] conducted comparative studies of single- and multi-layer fabrics, developed with the use of conventional and/or the latest technology, i.e., nano-nonwoven fabrics, while materials containing nano-nonwoven fabrics were included in the group of multi-layer fabrics. One fabric consisted of layers, which consisted of: *m*-aramid yarn based on the filament twill technology and fabric made of para-aramid yarn, polytetrafluoroethylene (PTFE) coated membrane on aramid nonwoven fabric, *m*-aramid nonwoven fabric, *m*-aramid nano nonwoven fabric, fabric with composition 93% *m*-aramid, 5% *p*-aramid, and 2% antistatic fiber. The second fabric, on the other hand, was made of layers consisting of a nonwoven fabric made of 75% *m*-aramid/23% *p*-aramid/2% antistatic nonwoven fabric, a PTFE-coated membrane on the aramid nonwoven fabric, a *m*-aramid nonwoven fabric, and a nano-nonwoven fabric [48]. The basic properties of these fabrics, such as weight, thickness, heat resistance, air permeability, resistance to evaporation, and water release rate, were measured using standard test methods developed by ISO or the AATCC (American Association of Textile Chemists and Colorists). On the other hand, the effectiveness of the thermal protection was measured under the conditions of exposure to flame and radiant heat of different intensities. The weight and thickness of fabrics containing nano-nonwoven fabrics classified these materials in the group of the three thinnest and five lightest fabrics. In addition, in the case of the values of parameters such as thermal resistance (°K·m^2^/W·10^−3^), air-permeability (cm^3^/cm^2^/s), and water (sweat) spreading speed (mm/s), these fabrics were in the group of five with the best parameters. These fabrics were the worst in the case of the parameter relating to evaporative resistance (m^2^·Pa/W), for which the values were 14.2 and 13.0, respectively, while the range of values for the group of multilayer fabrics was 9.4–25.4. The thermoprotective and thermophysiological properties of fabrics containing nano-nonwoven fabrics indicate that these fabrics meet the requirements in these areas [48].

Lessan et al. [49] developed a new fire retardant that could be applied to cotton fabrics. The agent was obtained from sodium hypophosphite, maleic acid, and triethanolamine, as well as TiO_2_ nanoparticles, while maleic acid oligomers were obtained by radical polymerization reactions. The bond between the oligomeric species of maleic acid and cellulose obtained as a result of the conducted processes is resistant to hydrolysis, which translates into greater durability of the material in the event of repeated washing at home [50,51].

Aluminum silicate nanofillers are gaining more and more popularity, including montmorillonite, hectorite, bentonite, and saponite, which, with appropriate dispersion, improve the mechanical properties of polymeric materials, increase resistance to inflammation, and increase the barrier to chemical substances [52]. The use of layered nanofillers allows one to obtain polymer intercalation nanocomposites and exfoliation nanocomposites. In the first case, the nanofiller plates are separated by single polymer chains and retain their layered structure, while in the second case, the nanofiller is evenly distributed in the polymer matrix [52].

The demand for functional and cost-effective flame-resistant textiles (FRT) is increasing, which is why Nie et al. [53] conducted research on the development of a simple casting method for the production of flame-resistant gel/textiles (FR-GT) composite materials based on acrylamide (AAM) and SiO_2_. The obtained results showed that the active diffusion of the aqueous pre-AAM/SiO_2_ gel to the textile structure enabled the formation of strong interfacial adhesion between the hydrogel and the textiles. The presence of the chemical cross-linking agent polyethylene glycol diacrylate (PEGDA) and the physical cross-linking agent SiO_2_ limited the expansion of the hydrogel volume during swelling. Additionally, the hydrogel layer of the nanocomposite polyacrylamide (PAAM)/SiO_2_ prevented burning in high temperature environments, i.e., >100 °C, due to the removal of heat from the water during evaporation. The produced hybrid hydrogel-textile composites are used in the production of fireproof materials, including fireproof gloves [53], and life-saving materials such as fireproof blankets [54].

### 3.2. Respiratory System

In relation to PPE, components such as respiratory protection equipment play an important role in addition to appropriate clothing. Applying the achievements of nanotechnology in these areas is also important. The filtering elements of respiratory protection, based on fibers made of polymers, perform their basic function of purifying the air from non-biological particles (i.e., aerosols such as dust, fumes, and mists). The development of solutions based on nanotechnology in respiratory protection equipment is aimed at improving parameters in the field of bioactivity, the self-cleaning of filter layers or activated carbon, extending the protective time of filters, the possibility of indicating the degree of loss of protective parameters, or the moment of penetration of the protective layer of the sorption bed, and in the case of absorbers which purify the air of specific organic vapors and gases generated in risk areas [52,55].

The self-cleaning effect of the aerosol particles embedded in the filtration structure or the molecules of organic substances adsorbed on the surface of active carbon can be achieved by using n-TiO_2_. Nanoparticles introduced into the structure or applied to the surface of polymer fibers forming the filter material, or bonded to the surface of the activated carbon at the appropriate humidity of the system and under the influence of UV radiation, make the filtering or absorbing medium self-cleaning [56]. Another important issue is the emerging threats of a biological nature (bioaerosols); therefore, the protection measures must be characterized by targeted bacteriostatic or bactericidal activity. In order to develop this type of filter material, it is necessary to introduce particles with the assumed biological activity into the structure of the polymer fiber. For this purpose, silver, copper, and gold nanoparticles are used [57].

The use of innovative techniques for modifying materials used to protect the respiratory system, such as electrospinning or the use of low-temperature plasma, make it possible to obtain new nanometer-sized filter fiber structures. The use of these types of fibers is related to the ability to effectively and permanently retain nanoparticles in a filter layer made of these types of fibers. The electrospinning technique also allows for the production of sensory fibers, making it possible to use them in filter materials as sensors capturing specific air pollutants, e.g., organic vapors, metal dusts [58,59]. In the case of modification of polymer fibers based on the low-temperature plasma technique, it allows one to not only to shape the physical structure of the fiber surface, but also to modify the chemical structure. By appropriate selection of the process parameters, e.g., the type of carrier gas, time, and power of interaction, it is possible to create nano-layers on the surface of the fibers, which determine the properties of the fiber in the area of hydrophobicity, hydrophilicity, or oleophobicity [60].

The use of plasma to chemically modify the surface of the activated carbon granules allows one to give specific properties to carbon sorbents. It allows the introduction of metal nanoparticles to the surface of the carbon granule, which allows for changes in conditions, e.g., pH or electroconductivity. Such effects make it possible to use modified carbon granules as an absorber bed as a sensor for organic vapors and gases, ammonia vapors, or acid vapors [52,61].

The hazards of wilderness fires vary widely. Heat, moisture, thermal radiation, incandescent particles, microparticles, nanoparticles, fog, water dispersion, viruses, bacteria, enzymes, proteins, or the presence of organic and inorganic gases and vapors, acids, amines, carbon monoxide, formaldehyde, acrolein, and volatile organic compounds (VOCs) pose a significant threat to the safety of firefighters. Therefore, work is underway to develop a permanent filter capable of removing carbon monoxide, formaldehyde, VOC, or NO_x_. For example, Vallfirest and Astrea Materials [62,63] used nanotechnology to develop a CO filter capable of effectively protecting wilderness firefighters during extinction operations. The product consists of a patented noble metal catalyst and an additional P3 R particle filter. Gold nanoparticles have the ability to eliminate pollutants such as CO, NO_x_, and formaldehyde at room temperature and in the presence of moisture (>90%) without the need for any drying step which would otherwise be required for conventional materials such as hopcalite. The cartridge is completed with a certified P3/P100 particle filter. The presence of a bayonet allows for direct connection to the Xtreme mask, guaranteeing good sealing and comfortable use during a fire, it is also compatible with all types of glasses, hearing protectors, and most helmets available on the market. The results of the fire test show that in a typical shift of 6 h, the filtration has an efficiency of >98% for CO and NO_x_, and >98% for the removal of formaldehyde, which is degraded to CO_2_ and H_2_O. Additionally, the developed nanomaterial has protective properties against VOCs [62,63].

## 4. Nanostructures in Tools in Fire Protection

Early detection of a fire, preferably in its initial stage, is essential in firefighting operations. The development of tools used for monitoring and signaling the threat aims to produce instruments and methods that will allow the detection of very low concentrations of smoke particles, temperature rise, light emission, or characteristic gaseous combustion products. It should be noted, however, that the emission of smoke, which is a colloidal system in which the dispersed phase is a liquid or a solid, largely depends on the properties of the material, the method of its combustion, and the stage of fire development. One of the basic elements of smoke detectors is a stable light source. Moreover, depending on the method of smoke detection, scattering or absorption, a radiation source of different wavelength and intensity is required. Nanotechnology allows one to obtain photoelements, the parameters of which significantly exceed conventional materials. The use of photoluminescent elements based on QD-LED quantum dots (Quantum Dot Light Emitting Device) allows one to increase the sensitivity. Such light sources are characterized by high stability, a narrow range of emitted light, and the possibility of wavelength adjustment, color purity, and processability of the solution [64].

### 4.1. Smoke Detectors

Smoke detectors also have a light sensor which uses various types of photoelements, such as photodiodes. By using nanowires based on gallium arsenide (GaAs) [65], graphene [66,67], or other materials [68,69,70], the sensitivity of light detectors as well as entire smoke detectors can be increased. The sensitivity of the detector based on GaAs/AlGaAs nanowires is 7.2 × 1010 cm·Hz1/2/W at a wavelength of 855 nm. This value is comparable to the high surface area gallium arsenide sensors [65,67]. In the research phase there are devices called nantennas, which are a nanometric version of a regular radio antenna, as a result of their size they are able to absorb light waves [71]. More developed versions of the device are based on the interaction of gold particle plasmons with the graphene plane, which makes it possible to detect light with extremely high sensitivity and in the frequency range that was previously characterized by low efficiency, i.e., infrared [72].

Fire analysis shows that smoke emission is lower during flame combustion for most materials. In the event of a flame, the thermal decomposition products are oxidized to a gaseous form, thereby reducing the amount of smoke emitted and increasing the amount of gases. The least noticeable method of combustion is oxygen, in the course of which the products of thermal decomposition are emitted. The smoldering material may remain unnoticed for a long time; therefore, it is important that even with minimal amounts of combustion products their identification and appropriate signal takes place. There are two primary exhaust gases in the air that are not normally present in high concentrations in, namely CO_2_ and CO. Since CO is not normally present in the air, its presence is a direct indicator of a fire. In practice, carbon monoxide detectors are used as sensors for alarming faulty ventilation. The most commonly used materials for the production of gas sensors include polymers, semiconductor metal oxides, or porous silica, and nanomaterials such as single-wall carbon nanotubes, graphene, and silicene [73].

Sensors based on carbon nanomaterials allow the detection of various gases, including CO, at the level of individual molecules [74,75]. The device analyzes the electrical properties of the nanoparticle connected to the electrodes, therefore, after adsorbing a given particle, e.g., CO, the electrical properties of the device change. Carbon nanotubes and 2D particles in the form of nanotubes or graphene are most often used for this purpose [76,77]. However, due to the weak interaction of the carbon structure with carbon monoxide, the range of detection of devices based on carbon tubes is limited [78]. Therefore, research was conducted on the use of the silicon equivalent of graphene, i.e., silicene. Silicene, due to slight interactions based on chemisorption, shows an increased sensitivity with respect to CO. In addition, heating of the CO molecules causes their desorption from the surface, regeneration, and the possibility of reusing the sensor. It should be noted, however, that the sensor works properly in a nitrogen atmosphere, but the presence of oxygen and water vapor reduces its effectiveness [79]. Carbon nanotubes are functionalized or coated with an alkali metal or alkali salt to improve the durability, life cycle of materials, or reduce the operating voltage [80].

Carbon nanofibers (CNF) show great potential for detection. CNF surfaces modified with various materials, such as metallic nanoparticles (NP), NP metal oxides, alloys, silica, and polymers, allow for the extension of the use of carbon nanofibers. CNF-based nanomaterial sensors are used to detect various parameters such as pressure, stress, and combustion products [81]. Depending on the type of material, carbon nanofibers used as sensors are classified into five types:

- clean CNF.
- CNF loaded with metal nanoparticles (NPs).
- CNF loaded with metal oxides nanoparticles (NPMOs).
- CNF loaded with metal alloys.
- other [81].

Li et al. [82] developed one-dimensional CNFs composed of graphite nanobrows using a simple electrospinning-assisted solid-phase graphitization method. The graphite CNF prepared in this way is sensitive to CO, and the detection limit for CO gas is only 50 ppm. It should also be added that the sensor enables the detection of other gases at room temperature, including H_2_, CH_4_, and ethanol [82].

The environmental and human health risk is related not only to the emission of combustion products, but also to the leakage of hazardous gases. Therefore, it is necessary to develop sensors that allow the detection of such gases as ammonia or hydrogen sulfide. Zhang et al. [83] developed a sensor based on ZnO-CNFs composites, allowing the detection of H_2_S at the level of 50–102 ppm [83]. Claramunt et al. [84] developed a sensor for the detection of ammonia. For this purpose, nanoparticles of the alloy of selected metals were deposited on CNF [84]. The sensitivity of CNF to which Au and Pd nanoparticles have been deposited show different levels of NH_3_ detection, depending on the percentage of individual nanoparticles. A sensor designed in such a way allows one to obtain a response within 5 min in the range of 110–120 °C. However, compared with a spectroscopic sensor, such as the mid-infrared sensor or the quartz-enhanced photoacoustic sensor [81], which have the advantages of rapid detection at room temperature without any reagent, the working temperature of Au and Pd NP-embellished CNFs is much higher. In order to lower the ammonia detection temperature, Lee et al. [85] modified CNF with WO_3_ nanoparticles, and as a consquence a detector allowing surface detection was obtained, the detector also had the possibility of detecting gaseous NO_2_ at room temperature, with a detection limit for NO_2_ at the level of 1 ppm [85].

Composite nanofibers (NF) are characterized by a large surface area and a variety of grain boundaries. These features allow NF to be used to develop highly sensitive and selective gas sensors. Gas detection properties can be significantly improved by modifying NF. The conductive channel and surface properties are adapted by modifying the composition using the synergistic effects of different materials and creating heterointerfaces. In the literature, one can find many works focusing on the synthesis of complex systems: NF-nanoparticles of selected metals/metal oxides by the electrospinning method (ES) [86]. For CO detection at the level of 0.1 or 1 ppm, CuO-ZnO [87] and CnO_2_-rGO [88] nanoparticles combined with NF were used, respectively, while for propane detection, the *p*-La0.67Sr0.33MnO_3_/n-CeO_2_/NF nanocomposition was used [89].

The sensors also use nanotubes or nanowires made of compounds such as TiO_2_, Si, or ZnO, functionalized or coated [90]. Nanowire detectors, obtained from the controlled ZnO suspension and deposition processes, can detect UV light with ten thousand times greater sensitivity than other zinc oxide detectors [91]. Photoelectric smoke detectors are able to detect larger particles in the case of denser smoke, while in the case of the emission of smaller particles, typical of fast-burning fires, their detection is delayed in time. As a result of their increased sensitivity, the new ZnO detectors are able to detect particles emitted specifically during the initial stages of a fire [80,92], which can significantly affect the reaction time and make it possible to save lives or reduce material damage caused by fires.

It should be noted, however, that the change of materials that make up the building elements, construction elements, or interior furnishings and decoration, causes changes in the spread of fires, as well as in the structure of sensors and the possibilities of detecting the emitted compounds.

### 4.2. Extinguishing

In order to combat fires, a number of fire suppression systems have been introduced that rely on various nano-scale chemical mixtures in order to effectively extinguishing fires [93,94]. Extinguishing agents affect combustion in many ways and therefore they are classified as: cooling, thinning, insulating, and inertial [95], with the most effective substances extinguishing the fire in several ways. The effectiveness of dry powders depends on their composition and particle size. The use of very fine and nano-sized particles of inorganic oxides is widespread among all-purpose firefighting agents for fires caused by combustible solids (class A), flammable liquids (class B), and gases (class C) [94,96,97].

Class A fire extinguishers include dry powder extinguishers based on ammonium phosphate and ammonium sulphate [98,99]. Two possible modes of action for such compounds are heterogeneous removal of reactive species and uniform inhibition. In addition to aluminum and magnesium hydroxides, zinc chloride, ferric hydroxide, sodium tetraborate and others are used as flame retardants for plastics and as extinguishing and firefighting agents [100,101]. Silica is also widely used in firefighting due to its large surface area and water absorption capacity. The use of silica allows the synthesis of ultrafast gelled nanostructured foams [102]. Foams cover the burning surfaces and isolate them from the spreading flame front. The foams separate the fuel from the oxygen, and the low-expansion foams provide significant cooling [94,96,103]. In recent years, a lot of work has been carried out on the preparation and application of fine and ultrafine extinguishing powders in order to determine the effect of particle size on the extinguishing efficiency and explosion protection. One of the best halon substitutes in many powder media is ammonium phosphate. Zhao et al. [104] showed that the extinguishing efficiency of ultra-fine dry ammonium phosphate powder with an average diameter of 11 μm improves with increasing injection pressure.

Hu et al. [105] investigated the reaction mechanism and thermal decomposition products of ammonium dihydrogen phosphate in the presence of ultrafine Mg(OH)_2_. It was found that the intermediate produced as a result of the reaction combine with each other, resulting in the formation of new extinguishing substances, which accelerates the extinguishing process. Thus, the extinguishing effect of the composite dry powder, which also includes Mg(OH)_2_, is higher than that of the powder extinguishing agent itself [105].

Among the available solutions and substances used as extinguishing material, powders are a universal solution. Moreover, they are characterized by high efficiency, longer service life, and a wider scope of application at low production and operating costs. It should also be added that powders as extinguishing agents do not cause significant damage to surrounding objects, so they can be used on many different objects [106].

Fire extinguishers are examples of innovative solutions using nanotechnology mainly for a comprehensive approach to firefighting. The mechanism of action of the extinguishing agents, however, may vary. The extinguishing effect can be obtained by cutting off access of oxygen, removing heat from the burning surface, and inhibiting radical reactions taking place in the flame. Due to the state of aggregation, the following extinguishing agents can be distinguished: liquid, foam, powder, and gas extinguishing agents. Not all substances will be effective in extinguishing a given fire. In addition to water, powders are among the most commonly used extinguishing agents. Their grain diameter ranges from 20 to 60 μm, and their action mechanism is based on the inhibition of combustion processes. The atomized powder falls onto the surface of the burning material and cuts off the access of oxygen by forming a glassy film, as is the case with phosphate powders. In addition, carbonate-based powders emit CO_2_ and water vapor as a result of thermal decomposition, which displaces oxygen. Extinguishing powders are also used to suppress explosions, where, when applied to the combustion zone, they prevent an explosion from developing to a significant size [94,107].

It should be noted that typical extinguishing powders are not very effective in extinguishing a gas flame because their contact surface and sedimentation time are not sufficient. Nanopowders with a grain diameter of approximately 100 nm fall in the air at a speed of 7.3 cm/day, and their specific surface area is on the order of 25–100 m^2^/g, thus their quenching efficiency is about 30 times higher than that of conventional powders [94].

Mosina et al. [108] used finely dispersed Al_2_O_3_ nanoparticles for extinguishing fires. Based on the results, it was found that nanocolloids based on aluminum hydroxide as extinguishing compositions can be used for two purposes: fire prevention and fire extinguishing with the creation of an aluminum oxide barrier. Aluminum sulphate and hydrolysing agents, including ammonia solution or sodium bicarbonate, were used in the production of hydrolates. As a result of heat treatment during combustion, a ceramic coating based on the γ-Al_2_O_3_ phase with a specific surface area of up to 150 m^2^/g was created. The resulting barrier made it possible to limit the access of oxygen while removing excess heat. An important piece of information is that the preparation of finely dispersed nanoparticles of inorganic oxides is possible directly at the fire site by the firefighters themselves. In addition, the resulting ceramic foam maintains stable, environmentally friendly properties and prevents re-ignition [108].

While these inorganic compounds are effective and recommended for use as additives in multi-component fire extinguishing compositions, they are not used independently. In addition, by themselves or as part of a composition, they can also be toxic to the environment. Against the background of other inorganic compounds, aluminum hydroxide shows potential for the formation of a protective film with exceptional heat resistance, mechanical strength, high biocompatibility, and insulating properties. The extinguishing capacity of dispersed nanoparticles is described as a function of the square root of their dispersion; therefore, the use of inorganic sols increases the effectiveness of the extinguishing agents [94].

As part of the research, work was also undertaken to reduce the particle size of dry extinguishing agents to about 100 nm. Particles of this size generate momentum which, during the extinguishing process, pushes the particles directly into the fire. Thus, the process carried out in this way reduces the number of residues after eliminating the fire. This type of work allowed for the development of portable fire extinguishers that are environmentally friendly and non-toxic. They can be used for fire, polyester materials, or a starting fire of electrical devices [109].

However, it should be remembered that not all nanomaterials will have a quenching effect. Some powders, such as NiO, exhibit a catalytic effect, thus accelerating combustion. ZrO_2_ works well for extinguishing purposes, as it has been proven to inhibit methane combustion. In addition to free fall and inhibitory properties, nanopowders, like any nanomaterial, are also characterized by high efficiency. Due to the large specific surface area, a small amount of powder can form an aerosol cloud that is effective in extinguishing the flames. Another solution is the use of microcapsules containing an active extinguishing agent. Under normal conditions, capsules of 10–50 μm in size behave like ordinary powders, and therefore do not have any chemical influence on the environment. In conditions of increased temperature, they burst and release the extinguishing agent directly in the combustion zone. This solution makes it possible to reduce the extinguisher to a handy size of approximately 20 cm [94]. In addition, its simple construction allows it to be used without the need for service for a period of 5 years, unless otherwise provided for in the regulations.

## 5. Nanoadditives in Industrial Products

The use of carefully selected additives allows for a significant increase in the fire resistance of materials used in the production of building and construction products, protective coatings, paints, textiles, and powders, which is of great importance in fire protection. A significant influence on the increase of thermal resistance and the related fire resistance is the incorporation of nano-additives into the structure of these materials such as carbon and halloysite nanotubes, nanoparticles of silica or zirconium, aluminosilicate nanofillers and others. The role and mechanism of action of nanoadditives depends on many factors, and above all on their morphology, the presence of functional groups and their thermal resistance. Nano-additives are usually classified according to their morphology as one-dimensional (including nanotubes, nanowires, and nanofibers), two-dimensional (including nano-sheets and wafers) and three-dimensional (such as spherical nanoparticles, nanoscopes, and nanoclusters). The morphology and functionalized of these nanoadditives have various effects on the thermal properties and fire resistance of the modified materials (Table 2).

An important factor influencing the properties of nanocomposite materials is the appropriate modification of nanoadditives, enabling the permanent incorporation of the nanoadditives into the structure of the nanocomposite. The selection of the appropriate method of modification of the nanoadditive depends on the morphology and chemical structure of the composite matrix, e.g., on the presence of functional groups that enable the formation of permanent bonds as a result of reacting with the functional groups of the nanoadditive. A significant part of the discussed composite materials, such as construction materials, protective coatings and membranes, paints, and textiles, are produced with the use of polymeric materials. The properties of the polymer matrix determine the selection of the most appropriate nanoadditives both in terms of their reactivity and structural properties enabling good dispersion and incorporation into the matrix structure.

Generally, nanoadditives used in plastics technology can be divided into three groups according to their influence on the thermal properties of polymer composites: non-hydrated, water-releasing, and functionalized. Non-hydrated nanoadditives improve the thermal resistance of silicone composites, while nanoadditives that release water from the processes of crystallization or condensation of hydroxyl groups reduce the thermal properties of these composites [118]. For example, the presence of hydroxyl groups in a nanoadditive, e.g., hydrophilic nanosilica, can lower the temperature of thermal degradation of silicone rubber through surface catalysed attack of hydroxyl groups [116]. Nanoadditives with a layered structure with lamellar particles should be modified in a way that enables effective exfoliation and intercalation in the structure of the nanocomposite [119]. Similarly, nanoadditives containing fibrous particles can be properly oriented during processing, positively influencing the properties of polymer composites [120]. These issues will be discussed later in this chapter for the different types of nanoadditives.

### 5.1. Carbon and Halloysite Nanotubes as Nanoadditives

The use of a nanoadditive in the form of carbon nanotubes, especially double-wall carbon nanotubes (DWNT), allows one to obtain polymer nanocomposites with significantly higher fire resistance compared to the properties of unmodified polymeric materials. The increased fire resistance of nancomposites containing carbon nanotubes is attributed to the formation of a continuous carbon layer that can act as a heat shield [121]. Moreover, the flame-retardant efficiency clearly depends on the degree of dispersion of the nanotubes and their content in the polymer matrix. In general, introducing about 0.5 wt.% gives the desired effect [122]. The introduction of DWNT into the silicone matrix allows for a significant increase in the fire resistance of the obtained nanocomposite, which makes it possible to use it as a coating material in aeronautics [111]. The best results were obtained with the use of a mixture of DWNT with carbon nanotubes in the amount of 0.25–0.5% by weight. In the case of the silicone matrix, better results were obtained with the use of unmodified nanotubes, which is explained by the increased affinity of such nanotubes for the silicone matrix. The addition of halloysite nanotubes also has a positive effect on the fire resistance of polymer composites. In order to obtain good miscibility with the polymer matrix, they require modification of the surface properties, most often with the use of (3-aminopropyl) triethoxysilane. The increase in fire resistance is in this case explained by the creation of a thermal barrier and the accumulation of degradation products of the polymeric material, e.g., silicone rubber inside the halloysite nanotubes [113].

### 5.2. Nanoparticles of Silica as Nanoadditives

Silica materials of various structures are widely used fillers for polymer composites and paints. In recent years, nanoparticles of silica (also commonly known as nanosilica) have been used more and more widely. An advantage of this is that it is possible to obtain the desired effect of improving properties by using them in a much smaller amount compared to conventional silicas. The method of introducing these nanosilicas into the polymer matrix is of significant importance for obtaining the desired increase in fire resistance. Hydrophilic nanosilica has reactive silanol groups on its surface that enable the incorporation of nanoparticles into the polymer matrix. However, when a silicone matrix is used, the presence of silanol groups can cause significant processing difficulties due to the formation of hydrogen bonds between the oxygen of the organosilicon polymer chain and the silanol groups of the silica. This can make the processing of the polymer composite much more difficult [123]. In order to avoid these difficulties, it is preferable to use hydrophobized nanosilica with blocked hydroxyl groups on the surface. Regardless of the type of nanosilica, it is necessary to disperse the nanoparticles well in the polymer matrix because the mechanism for increasing the fire resistance of the polymer nanocomposite due to the introduction of nanosilica is related to physical processes taking place in the solid phase and not to chemical reactions [115]. Therefore, good dispersion of nanosilica in the polymer matrix has a significant impact on obtaining the desired properties, including fire resistance. The influence of nanosilica particle size on fire resistance was also confirmed. Nodera and Kanai [124] found that nanosilica with a nanoparticle size of 20 nm incorporated into the PC/PDMS composite (Polycarbonate/Polydimethylsiloxane) in an amount of 0.5 wt had a greater effect on fire resistance compared to nanosilica with a particle size of 50 nm introduced in the same amount. In recent years, there has also been a great development in the field of polysilsesquioxanes (POSS), the main advantage of which is a very well-defined structure and chemical composition [125]. The possibility of introducing various functional groups into the POSS structure, ensuring their chemical bond with the polymer matrix, allows one to obtain the desired properties of polymer nanocomposites that remain unchanged during use [126]. Moreover, synergistic effects may occur between POSS and nanosilica, allowing for the production of polymer composites with very good properties [127]. Currently, the price of these nanofillers is still the main barrier hindering the widespread usage of POSS [125].

### 5.3. Substances with a Layered or Coniferous Structure as Nanoadditives

Nanoadditives with a layered structure enable an increase in fire resistance of polymer composites. This group of nanoadditives includes not only montmorillonite, but also kaolin, talc, and mica, which are widely used in the processing of polymeric materials. The positive effect of introducing these additives is obtained after their proper dispersion in the nanocomposite structure and is related to obtaining nanolayer structures. Appropriate chemical modification of these fillers facilitates the exfoliation and intercalation of these nanolayers [128]. Based on the results obtained by various research teams, it can be concluded that layered nanoadditives are capable of creating a silica-carbon sinter layer during the combustion of a polymer nanocomposite [129]. This effect is particularly evident in polymer nanocomposites containing organosilicon polymers, such as polydimethylsiloxane (PDMS), which is commonly referred to as silicone rubber [114]. It was found that the formation of a silica-carbon sinter layer occurs particularly clearly in nanocomposites containing montmorillonite. Complex processes take place during the combustion of such composites, and the formation of the sinter increases the fire resistance due to the mass [130,131] and thermal barrier effect [132], the labyrinth effect [133], and the steric [134] and catalytic effect [135]. The massive barrier effect is due to the limitation of diffusion of the nanocomposite combustion products through the sinter layer. Moreover, some of these products can be incorporated into the sinter layer, which is also advantageous in that it reduces the amount of secreted, often toxic, combustion products. The thermal barrier effect is related to the insulating of the interior of the nanocomposite by the sinter layer, which reduces the weight loss of the nanocomposite during combustion. In addition, the sinter contains montmorillonite nano-layers which form the labyrinth, thus hindering the access of oxygen to the inside of the sample, making it difficult to burn. Intercalated montmorillonite nanolayers limit the mobility of polymer chains and have a catalytic effect on the formation of the sinter. The remaining nanoadditives with a layered structure are introduced into polymer nanocomposites in order to obtain a more solid sinter structure. For example, the inclusion of kaolin in a PDMS-based composite enables a frit with fewer cracks to be obtained [136].

The most widely used nano-additive with a needle structure is wollastonite, which is one of the crystallographic forms of calcium silicate. This nano-additive is often used as a component of ceramizing silicone rubber mixtures. It has been extensively tested and patented for electrical wire sheathing [137,138]. The best results are obtained with the use of carbofunctional silane modified wollastonite, e.g., (γ-vinylpropyl) triethoxysilane. The use of a carbofunctional silane improves the adhesion of the nano-additive to the polymer matrix by creating chemical bonds between the hydroxyl groups on the filler surface and the ethoxy groups of the carbofunctional silane. The vinyl group is incorporated into the structure of the polymer matrix during the cross-linking process of the vinyl siloxane rubber.

### 5.4. Examples of the Use of Nanoadditives in Industrial Products

The continuous development of innovative material and technological solutions enables the development of materials and products characterized by increased fire resistance. The use of such products significantly increases the safety of users during a fire. One example is fire-retardant coatings which are increasingly used because of their unique properties, especially related to their very good resistance to high temperatures. In recent years, there has been a great development in the field of intumescent coatings and paints used to protect steel structures. Such structures require protection with appropriate coatings or other construction solutions in order to guarantee safety in the event of a fire. The proper selection of fillers [139], including nanoadditives, is essential for obtaining the desired fire resistance of protective coatings and intumescent paints, such as aluminosilicate nanofillers: montmorillonite (MMT) and sepiolite [140], zirconium nanoparticles and chitosan [141], nanosilica [142], carbon nanotubes or POSS [143], nanosilica, and chitosan [144]. The use of intumescent fire-retardant coatings is a very effective method of protecting materials, which can be used both for metal surfaces as well as for plastics, steel, wood, electric cables, and polymer composites [145,146]. The intumescent fireproof coating obtained from a silicone-acrylic binder with the addition of nanosilica has been used to protect a tunnel [147]. The possibility of using such coatings based on an epoxy-silicone binder for the protection of polycarbonate sheets has also been confirmed [148]. The applied intumescent protective coating also limited the dripping of the material during a fire.

By analyzing the available data [149], it can be concluded that properly selected nanoadditives can result in better fire resistance in polymer composites and can significantly increase the smoke suppression effect during their combustion. In addition, in order to improve fire resistance, it is also important to control the degree of dispersion of nanoadditives in polymer matrices [150]. The large interface between the nano-additive and the polymer can enhance catalytic effects such as the catalysis of the carbonization reaction or radical scavenging processes. The formation of a continuous protective layer consisting of a network of nanoparticles has been found to be a key flame retardant process for layered nanoadditives, where the layer appears to act as a physical shield [151]. Creating such a network is also important for improving other physical properties of polymer composites, which continues to be a challenge. If the nano-additive does not have a sufficient effect on fire resistance, a good solution is to use it together with other flame retardant additives, the so-called flame retardants.

## 6. The Hazards of Nanosubstances

An important issue in the field of nanotechnology is the possibility of emission and negative impact on the human body of nanoparticles. The work carried out in the field of combustion products on materials containing nanoparticles indicates that nanoparticles with the same or different properties can be formed. Metal nanoparticles, which are widely used as modifiers or fillers, may be released into the atmosphere during combustion processes, determining environmental processes and affecting human health. For example, the main combustion products of PDMS are SiO_2_, CO_2_, and H_2_O. Silica (SiO_2_) creates dust with excellent dielectric properties and provides additional protection against further fire development [152]. On the other hand, the emission of nanoparticles can pose a significant threat. For example, in the event of a fire in a store with sports goods, including carbon fiber items such as skis, or a store with household appliances, where there are flat-screen TVs or powder-coated items containing nanoparticles, the combustion process emits carbon nanoparticles, metals and their compounds.

A large amount of nanoparticles can also be emitted into the air from waste and materials that arise in industrial areas. Metal nanoparticles (NPMs) are used, among others in the construction and textile industries. Materials and substances constituting post-production waste or waste due to damage or defect are collected and stored in places that are often discovered, as a result of this they are exposed to the effects of various weather conditions, such as temperature, precipitation, and radiation. The data show that the amounts of waste generated by the production processes are in a few cases significantly greater than the amount of the final product of NPMs. In the event of damage to the packaging, it is necessary to manage it through storage or recycling/utilization, depending on the type of waste. When combustion processes are used, nanoparticles are released into the air. NMPs emissions may also occur during waste collection as a result of the abrasion process during mechanical compaction. During recycling processes, the destruction and sorting of materials may require mechanical shredding. The actual release to the environment can, therefore, not only take place “within” an actual waste treatment facility, but also in the management of residual streams from waste facilities. For example in the treatment of flue-gas treatment residues, or in the management of sludge from leachate treatment from landfills.

Safety concerns arise when nanocompounds have an impact on human health and/or the environment or pose a fire and explosion hazard, with information on their lower explosive limit [57]. Airborne nanoparticles enter the bloodstream 15 min after someone has been forced to inhale them. This is because nanoparticles are able to break the barrier between the lungs and the bloodstream, increasing the risk of a heart attack or stroke. Ag nanoparticles accumulate in the kidneys, liver, intestines, tongue, and brain, leading to cell death. They damage DNA and nerve cells, penetrate and damage the blood-brain barrier (BBB), produce free radicals, and cause inflammation [153,154]. After entering the body, iron nanoparticles also change the expression of genes, disrupt the functioning of cells leading to apoptosis, cause oxidative stress, and disturb iron homeostasis. They are believed to be the cause of Alzheimer’s and Parkinson’s disease [57,155]. Copper nanoparticles, which are commonly used in industrial materials to improve thermal properties, penetrate the blood-brain barrier, leading to the degeneration of the nervous system cells [156]. It should be noted, however, that copper oxide nanoparticles are more neurotoxic than other metals.

The primary way of absorption of nanoparticles into the human body is the respiratory system [157]. Particles suspended in the inhaled air enter the human body through the nose and/or mouth, and then may move through the successively bifurcating ducts of the bronchial tree to the respiratory part or be deposited in individual regions of the respiratory system. Studies carried out with the use of nanoparticles show that particles smaller than 10 nm are mainly retained in the upper respiratory tract. Particles with dimensions of 10–100 nm are deposited in the area of bronchioles and alveoli with an efficiency of 20–60%. Studies carried out with the use of carbon nanoparticles with dimensions below 100 nm show that only 25% of the deposited nanoparticles are removed from the respiratory system within 24 h. The retention time of 75% of carbon nanoparticles introduced into the respiratory system as a result of inhalation exceeds 48 h [158]. The long retention time of nanoparticles in the lungs promotes their penetration into the epithelial cells of the respiratory tract, bloodstream, or the lymphatic system. The widespread use of titanium nanoparticles and its compounds is of concern. Studies have shown that it crosses the blood-brain barrier and accumulates primarily in the hippocampus, which means that it is neurotoxic. After administration to female mice, it was found to accumulate in the nervous system of the fetus and to impede fetal development.

According to the Occupational Safety and Health Administration (OSHA), workers using nanomaterials during their research or production processes may potentially be exposed to nanoparticles through inhalation, skin contact, or ingestion, depending on material handling procedures [159]. It was also noted that nanosubstances can easily penetrate intact human skin and cause toxicological reactions in the lungs of exposed experimental animals [159]. The toxicity of engineered nanoparticles is similar to that of other chemicals used in the manufacturing industry. In addition, OSHA suggests that toxicity depends on the physical and chemical properties of the particle, such as particle size, physical state, shape, crystal structure, chemical composition, surface area, and surface chemistry, charge. It should be added, however, that in the case of nanoparticles, the surface area and the number of particles in the air volume (numerical concentration) are a better measure of exposure than the mass concentration. In addition, surface area is a parameter that plays a key role in the toxicity of nanoparticles. An important problem is the lack of normative or indicative values for the exposure assessment. In some countries, for example, in Great Britain, a pragmatic approach is proposed using the so-called dose/reference level as an acceptable exposure level [160]. According to EU-OSHA [161] recommendations, the amount of TiO_2_ nanoparticles in the air cannot exceed 0.3 mg/m^3^, while for particles larger than 100 nm, i.e., beyond the nanoscale, the amount is 2.4 mg/m^3^ NIOSH REL [161].

The development of safeguards and procedures is key to protecting humans from nanoparticles, unfortunately, largely due to a lack of specific knowledge about the effects of nanoscale materials on the human body, there are currently no specific medical evaluation protocols. Airborne nanoparticles are now treated in the same way as ultrafine particles. The research results also indicate the possibility of influencing the formation of neoplastic diseases. Tests on mice have shown that long and straight multi-walled carbon nanotubes can lead to the same type of inflammation and changes in the tissues surrounding the lungs, and the same effects of exposure as asbestos. Another study on mice found that similar carbon nanotubes eventually led to cancerous tumors. While there are different types of nano-substances, some can cause disease in those who both inhale the particles and are in their vicinity.

The presence of nanoparticles in the air can play a significant role in terms of fire safety. In industrial plants, metal nanoparticles can be released during various processes. The emission may come from powder, liquid, and solid matrix containing nanofillers (e.g., nanocomposites) [162]. Aluminum dust, which is generated during the processing or manufacturing of aluminum products, can generate suspended dust clouds. Violent explosions may occur if exposed to ignition sources with sufficient energy, such as electric sparks or hot surfaces. The test results indicate that the aluminum dust explosion temperature exceeding 2273 K (2000 °C), in combination with the excess pressure caused by the explosion, will cause significant damage [163]. Such a situation took place in Kunshan Zhongrong Metal Products Co., Ltd. in Kunshan, China on 2 August 2014. As a result of the disaster, 146 people were killed and 91 were injured [164,165].

The presence of nanoparticles in the air with magnetic and catalytic properties, such as Fe and Ti, which are the source of reactive oxygen species, can rapidly accelerate the reaction. Changes in the rate of oxidation, catalysis and other reactions taking place in the air should be taken into account in the models of pollutant migration [57,166]. For carbon, aluminum, and magnesium nanotubes, a significant correlation was found between the particle size and the explosion parameters. It was also noted that the particle size is important for understanding the explosion process in the presence of nanoparticles. It is also necessary to take into account factors such as concentration, turbulence, or the possibility of dispersion in a given center. This is all the more important as different results were obtained depending on the experimental conditions used (e.g., different concentrations). The chemical nature of the analyzed elements was also important. For example, the minimum explosible concentration (MEC) values indicate that aluminum nanotubes and magnesium nanotubes behave differently. A plateau was obtained for the aluminum particles, whereas the MEC value for the magnesium slowly increased to a certain point as the particle size increased, followed by a sudden increase. It was also found that because nanoparticles are characterized by a low bulk density, their thermal conductivity is low and they tend to self-heat when placed in containers. Therefore, it is necessary to define the conditions of their storage in order to minimize the risk [167]. It also seems necessary to start work on the role of metal nanoparticles in the area of faster combustion processes or fire hazards.

## 7. Conclusions

The use of nanotechnology made it possible to combine the requirements for resistance to external factors, such as high temperature. On the other hand, it allowed for the increased safety of firefighters during rescue and firefighting operations. The use of nanoporous aerogels, nanoclay-reinforced resin coatings on fabrics/nonwovens, nanofibers in nonwovens, nano-finishes in phase change materials, or shape memory alloys in nonwovens/nonwovens, make a significant contribution to improving the protective properties and comfort of materials used in clothing and protective equipment worn by firefighters. In addition, the implementation of intelligent technologies such as electric wires, sensors, gas detectors, security alarms, devices placed in protective clothing and equipment, can also significantly increase safety by monitoring work in difficult conditions. The new materials increase the sensitivity of fire detection systems and their reliability. Detectors based on nanomaterials allow the detection of thermal decomposition and combustion products with the accuracy of single molecules. New light sources and light sensors can increase the capabilities and reliability of smoke detectors. Nanotechnology also makes a significant contribution to the development of extinguishing agents. Modern extinguishing powders, as a result of their extremely small size, are more efficient than conventional powders.

Nanotechnology-based materials can also significantly improve fire protection and risk prevention strategies. The combination of solutions used in various areas of materials, construction, and monitoring with nanotechnology can strengthen the quality of actions taken at the preventive stage, and can improve the actions to minimize the risk. However, it should be noted that the development of new materials may also affect the conditions for the emergence and spread of threats, as well as the type of compounds emitted in the environment. Therefore, further research is needed on the application and the consequences and impacts of nanotechnology-based solutions on the environment and on the health and safety of firefighters.

## Figures and Tables

**Table 1:** Examples of fire protection requirements.

The Scope of the Requirements	Document	Characteristics	Ref.
Fire brigades/CA	OSHS, 1910.156	It specifies the requirements for, inter alia, organization, training, and personal protective equipment of the fire brigade	[10]
Protective clothing and equipment for firefighting/US	NFPA 1977	It specifies the minimum design, performance, testing, and certification requirements for elements of protective clothing and firefighting equipment in wild and urban areas, including protective clothing, protective helmets, protective gloves, protective footwear, goggles, and chain saw protectors; and for load carrying equipment.	[11]
Fire Code/US	NFPA 1	Refers to over 130 codes and standards of NFPA^®^, including, but not limited to, industry patterns; includes, among other things, inspections of permanent and temporary buildings, processes, equipment, inspection of construction plans, drawings, and specifications for safety systems.	[12]
Fire truck/US	NFPA 1901	Requirements for new automotive fire-fighting devices and trailers intended for the transport of personnel and equipment in emergency conditions.	[13]
Sprinkler System Installation/US	NFPA 13	It specifies the minimum requirements for the design and installation of automatic fire sprinkler systems; does not include requirements for the design or installation of water mist fire protection systems.	[14]
Production of organic coatings/US	NFPA 35	Used for facilities that use flammable liquids to produce organic coatings for automotive, industrial, institutional, home, marine, printing, transportation, and other applications; does not include, inter alia, spray application with combustible materials, dipping, coating, and printing processes using combustible liquids.	[15]
Prevention, preparedness, and response to chemical accidents/UE	CFPA-E Guideline No 18: 2013 F	It concerns the prevention of chemical accidents; application to buildings (plants) producing chemicals and define preventive and emergency measures that help reduce damage after a fire or explosion (including, inter alia, synthesis, physical operations such as formulation and standardization, in production and pilot plants); does not apply to warehouses, tanks, and laboratories.	[16]
Building sites/UE	CFPA-E Guideline No 21: 2021 F	Intended for construction works, including renovation; the target group are, among other clients, developers, contractors, emergency services, fire consultants, insurers; the guidelines apply to larger buildings, they do not concern fire protection problems and solutions for underground construction works.	[17]
Fire classification of construction products and building elements/UE	EN 13501–1: 2018	Classification of construction products in terms of fire behavior and fire resistance; the following are essential: maintaining the load-bearing capacity of the structure and fire spreading conditions; it is necessary to demonstrate the fire resistance of the load-bearing and/or room-separating building elements over time; introduces Euroclasses.	[18]
Fire protection products/UE	ETAG 018–1–3	Requirements for performance, criteria for assessing fire protection products in facilities; division into three groups of products and sets.	[19]
Basic protective clothing for a firefighter/PL	EN 469: 2020	Specifies the basic protective clothing for a firefighter, which is used during firefighting actions and related activities; deals with the layouts of fabrics used in garments, accessories, seams, and structures.	[20]
Protective clothing against chemicals/PL	EN 14325: 2018	Specifies test methods and classification of materials, seams, permanent and separable joints used in chemical protective clothing.	[21]

**Table 2:** The influence of morphology and functionalisation of nanoadditives on thermal properties and fire resistance of modified materials.

The Morphology of the Nanoadditive	Type of Nanoadditive	Modified Material	Thermal Resistance	Ref.
one-dimensional	modified halloysite nanotubes	polypropylene	increased	[110]
	modified carbon nanotubes with double walls	silicone rubber	reduced	[111]
	unmodified carbon nanotubes with double walls	silicone rubber	increased	[111]
	modified carbon nanotubes with double walls	poly(methyl methacrylate)	increased	[112]
	unmodified halloysite nanotubes	silicone rubber	reduced	[113]
two-dimensional	montmorillonite	poly(methyl methacrylate)	increased	[114]
three-dimensional	hydrophobized nanosilica	silicone rubber	increased	[115]
	hydrophilic nanosilica	silicone rubber	reduced	[116]
	hydrophilic nanosilica	polyethylene terephthalate	increased	[117]

## Abbreviations

AAM	Acrylamide
AATCC	American Association of Textile Chemists and Colorists
BBB	Blood-Brain Barrier
CCOHS	Canadian Center for Occupational Health and Safety
CEA	The European Insurance and Reinsurance Federation
CFPA	Europe—Confederation of Fire Protection Associations Europe
CNF	Carbon Nanofibers
DWNT	Double-Wall Carbon Nanotubes
EN	European Standard
EOTA	European Organization for Technical Approvals
ETA	European Technical Assessments
ETAG	European Technical Approval Guideline
EU	European Union
FPC	Firefighter’s Protective Clothing
FR-GT	Flame-Resistant Gel/Textiles
FRT	Flame-Resistant Textiles
LOI	Limited Oxygen Index
MEC	Minimum Explosible Concentration
MFB	Metropolitan Fire Brigade
MMT	Montmorillonite
MPIA	Poly(*m*-phenylene isophthalamide)
NFPA	National Fire Protection Association
NIOSH	US National Institute for Occupational Safety and Health
NPMOs	Metal Oxides Nanoparticles
NPMs	Metal Nanoparticles
NPs	Nanoparticles
OSHA	Occupational Safety and Health Administration
PAAM	Polyacrylamide
PC/PDMS	Polycarbonate/Polydimethylsiloxane
PCM	Phase-Change Materials
PDMS	Polydimethylsiloxane
PD-T, PPDT, or PPTA	Poly(*m*-phenylene terephthalamide)
PEGDA	Polyethylene Glycol Diacrylate
PMC	Polymer Matrix Composites
PN-EN	Polish Standard implementing the European Standard
POSS	Polysilsesquioxanes
PPD-T	Poly(*p*-phenylene terephthalamide)
PPE	Personal Protective Equipment
PTFE	Polytetrafluoroethylene
QD-LED	Quantum Dot Light Emitting Device
REI	Fire Resistance
REL	Recommended Exposure Limit
rGO	Reduced Graphene Oxide
SMM	Shape Memory Materials
VdS	VdS Schadenverhütung GmbH
VOC	Volatile Organic Compound

## References

[1] (2018). Safeopia, Fire Protection. https://www.safeopedia.com/definition/193/fire-protection.

[2] https://www.nfpa.org/.

[3] https://www.ccohs.ca/.

[4] https://www.pilz.com/pl-PL/support/knowhow/law-standards-norms/international-standards/north-america.

[5] https://www.eota.eu/.

[6] https://cfpa-e.eu/.

[7] PU Europe (2020). Fire Handbook, European Fire Standards, and National Legislation. http://highperformanceinsulation.eu/wp-content/uploads/2020/10/PU-Europe-Fire-Safety-Handbook-_-European-fire-standards-and-national-legislation-_June-2020.pdf.

[8] https://www.gov.pl/web/kgpsp-en.

[9] The Act of 24 November 1991–The Act on Fire Protection. No. 81, Item 351 (Dz. U. 1991 Nr 81 Poz. 351). Poland. https://isap.sejm.gov.pl/isap.nsf/DocDetails.xsp?id=WDU19910810351.

[10] OSHA. 1910.156-Fire Brigades. GPO Source e-CFR. Occupational Safety and Health Standards. December 2008. https://www.osha.gov/laws-regs/regulations/standardnumber/1910/1910.156.

[11] National Fire Protection Association (2021). NFPA 1977, Standard on Protective Clothing and Equipment for Wildland Fire Fighting and Urban Interface Fire Fighting.

[12] National Fire Protection Association (2021). NFPA 1, Fire Code.

[13] National Fire Protection Association (2016). NFPA 1901, Standard for Automotive Fire Apparaturs.

[14] National Fire Protection Association (2021). NFPA 13, Standard for the Installation of Sprinkler Systems.

[15] National Fire Protection Association (2020). NFPA 35, Standard for the Manufacture of Organic Coatings.

[16] The Confederation of Fire Protection Associations Europe (2013). CFPA E Guideline No 18 2013 F, Fire Protection on Chemical Manufacturing Sites.

[17] The Confederation of Fire Protection Associations Europe (2021). CFPA-E Guideline No 21:2021 F, Fire Prevention on Construction Sites.

[18] (2018). EN 13501-1:2018, Fire Classification of Construction Products and Building Elements-Part 1: Classification Using Data from Reaction to Fire Tests.

[19] (2013). ETAG 018, Guideline for European Technical Approval of Fire Protective Products.

[20] (2018). EN 469: Protective Clothing for Firefighters-Performance Requirements for Protective Clothing for Firefighting Activities.

[21] (2018). EN 14325: Protective Clothing Against Chemicals-Test Methods and Performance Classification of Chemical Protective Clothing Materials, Seams, Joins and Assemblage.

[22] Wfrgent Nv Global Safety Fire Resistance Classes. https://www.wfrgent.com/en/fire-resistance/fire-resistance-classes.html.

[23] (2016). EN 13501-2:2016 Fire Classification of Construction Products and Building Elements-Part 2: Classification Using Data from Fire Resistance Tests, Excluding Ventilation Services.

[24] (2020). Commission Regulation (EU) 2020/878 of 18 June 2020 amending Annex II to Regulation (EC) No 1907/2006 of the European Parliament and of the Council concerning the Registration, Evaluation, Authorisation and Restriction of Chemicals (REACH) *Off*. J. Eur. Union.

[25] Pavlicek A., Part F., Rose G., Praetorius A., Miernicki M., Gazsó A., Huber-Humer M. (2021). A European nano-registry as a reliable database for quantitative risk assessment of nanomaterials? A comparison of national approaches. NanoImpact.

[26] (2016). Regulation (EU) 2016/425 of the European Parliament and of the Council of 9 March 2016 on personal protective equipment and repealing Council Directive 89/686/EEC (Text with EEA relevance). Off. J. Eur. Union.

[27] Park H., Park J., Lin S.-H., Boorady L.M. (2014). Assessment of Firefighters’ needs for personal protective equipment. Fash. Text..

[28] Song G. (2017). Thermal Protective Clothing for Firefighters.

[29] Song G., Lu Y., Kilinc F.S. (2013). Flame resistant textiles for structural and proximity fire fighting. Handbook of Fire Resistant Textiles.

[30] Trigo-Lopez M., Garcia J.M., Ruiz J.A.R., Garcia F.C., Ferrer R. (2018). Aromatic Polyamides. Encyclopedia of Polymer Science and Technology.

[31] IMATTEC, International Materiaux et Textilles Techniques Aramids and Their Properties. https://imattec.com/en/aramids.php.

[32] Polymer Properties Database Aramid Fibers. Chemical Retrieval on the Web CROW 2015–2021. https://polymerdatabase.com/Fibers/Aramid.html.

[33] Morgan A.B., Kilinc F.S. (2013). Flame Retardant Fiber-Reinforced Composites. Handbook of Fire Resistant Textiles.

[34] Bourbigot S., Horrocks A.R., Price D. (2008). Flame retardancy of textiles: New approaches. Advances in Fire Retardant Materials.

[35] Mandal S., Camenzind M., Annaheim S., Rossi R.M. (2018). Firefighters’ Protective Clothing and Equipment. Firef. Cloth. Equip..

[36] Samanta A.K., Baghchi A., Biswas S.K. (2011). Fire retardant finishing of jute fabric and its thermal behaviour using phosphorous and nitrogen based compound. J. Polym. Mater..

[37] Tsukada M., Khan M.R., Tanaka T., Morikawa H. (2011). Thermal characteristics and physical properties of silk fabrics grafted with phosphorous flame retardant agents. Text. Res. J..

[38] Kundu C.K., Li Z., Song L., Hu Y. (2020). An overview of fire retardant treatments for synthetic textiles: From traditional approaches to recent applications. Eur. Polym. J..

[39] Shaid A., Wang L., Padhye R., Bhuyian M.A.R. (2018). Aerogel nonwoven as reinforcement and batting material for firefighter’s protective clothing: A comparative study. J. Sol-Gel Sci. Technol..

[40] Liu X.X., Lin L.T., Wang X.D., Zheng H.Q., Li Y., Luo X.N., Liu Y.F. (2011). Study on temperature response and thermal protection of shape memory com-bination fabrics. Textile Bioengineering and Informatics Symposium Proceedings 2011, Beijing, China, 27–29 May 2011.

[41] Kim H., Abdala A.A., Macosko C.W. (2010). Graphene/polymer nanocomposites. Macromolecules.

[42] Byrne M.T., Gun’Ko Y.K. (2010). Recent Advances in Research on Carbon Nanotube-Polymer Composites. Adv. Mater..

[43] Paul D., Robeson L. (2008). Polymer nanotechnology: Nanocomposites. Polymer.

[44] Ray S.S., Okamoto M. (2003). Polymer/layered silicate nanocomposites: A review from preparation to processing. Prog. Polym. Sci..

[45] Morgan A.B. (2006). Flame retarded polymer layered silicate nanocomposites: A review of commercial and open literature systems. Polym. Adv. Technol..

[46] Dadi H.H. (2010). Literature Overview of Smart Textiles. Master’s Thesis.

[47] Chitrphiromsri P., Kuznetsov A.V., Song G., Barker R.L. (2006). Investigation of Feasibility of Developing Intelligent Firefighter-Protective Garments Based on the Utilization of a Water-Injection System. Numer. Heat Transf. Part A Appl..

[48] Mandal S., Annaheim S., Greve J., Camenzind M., Rossi R.M. (2019). Modeling for predicting the thermal protective and thermo-physiological comfort performance of fabrics used in firefighters’ clothing. Text. Res. J..

[49] Lessan F., Montazer M., Moghadam M. (2011). A novel durable flame-retardant cotton fabric using sodium hypophosphite, nano TiO2 and maleic acid. Thermochim. Acta.

[50] Cheng X., Yang C.Q. (2009). Flame retardant finishing of cotton fleece fabric: Part, V. Phosphorus-containing maleic acid oligomers. Fire Mater..

[51] Joseph P., Tretsiakova-McNally S., Kilinc F.S. (2013). Chemical modification of natural and synthetic textile fibres to improve flame retardancy. Handbook of Fire Resistant Textiles.

[52] Krzemińska S., Hryny K.R., Pietrowski P. (2009). Possible application of nanomaterials in personal protective equipment. Work. Saf. Sci. Pract..

[53] Nie Y., Mugaanire I.T., Guo Y., Wang R., Hou K., Zhu M. (2021). A hybrid hydrogel/textile composite as flame-resistant dress. Prog. Nat. Sci..

[54] Illeperuma W.R.K., Rothemund P., Suo Z., Vlassak J.J. (2016). Fire-Resistant Hydrogel-Fabric Laminates: A Simple Concept That May Save Lives. ACS Appl. Mater. Interfaces.

[55] Niraj S., Jiazhi M., Yeow J.T.W. (2006). Carbon Nanotube-Based Sensors. J. Nanosci. Nanotechnol..

[56] Kim S.C., Kang S., Lee H., Kwak D.-B., Ou Q., Pei C., Pui D.Y. (2020). Nanofiber Filter Performance Improvement: Nanofiber Layer Uniformity and Branched Nanofiber. Aerosol Air Qual. Res..

[57] Rabajczyk A., Zielecka M., Porowski R., Hopke P.K. (2020). Metal nanoparticles in the air: State of the art and future perspectives. Environ. Sci. Nano.

[58] Zhang W., He Z., Han Y., Jiang Q., Zhan C., Zhang K., Li Z., Zhang R. (2020). Structural design and environmental applications of electrospun nanofibers. Compos. Part A Appl. Sci. Manuf..

[59] Wang S.-X., Yap C.C., He J., Chen C., Wong S.Y., Li X. (2016). Electrospinning: A facile technique for fabricating functional nanofibers for environmental applications. Nanotechnol. Rev..

[60] Vasile C. (2018). Polymeric Nanocomposites and Nanocoatings for Food Packaging: A Review. Materials.

[61] Qu G.-Z., Li J., Liang D.-L., Huang D.-L., Qu D., Huang Y.-M. (2013). Surface modification of a granular activated carbon by dielectric barrier discharge plasma and its effects on pentachlorophenol adsorption. J. Electrost..

[62] Vallfirest. Protection against CO, Formaldehyde, NOx, VOCs and Particles for Wildland Firefighters. Proceedings of the 16th International Wildland Fire Safety Summit, 6th Human Dimensions of Wildland Fire Conference.

[63] Xtreme Mask, The Latest Advance in Respiratory Protection for Wildfires (2020). Vallfirest–Wildland. News. https://wildlandfirefighter.com/2020/07/17/xtreme-mask-the-latest-advance-in-respiratory-protection-for-wildfires/.

[64] Wood V., Bulović V. (2010). Colloidal quantum dot light-emitting devices. Nano Rev..

[65] Dai X., Zhang S., Wang Z., Adamo G., Liu H., Huang Y., Couteau C., Soci C. (2014). GaAs/AlGaAs Nanowire Photodetector. Nano Lett..

[66] Klekachev A.V., Nourbakhsh A., Asselberghs I., Stesmans A.L., Heyns M.M., De Gendt S. (2013). Graphene Transistors and Photodetectors. Electrochem. Soc. Interface.

[67] Li J., Niu L., Zheng Z., Yan F. (2014). Photosensitive Graphene Transistors. Adv. Mater..

[68] Jiang C., Song J. (2014). Significant Photoelectric Property Change Caused by Additional Nano-confinement: A Study of Half-Dimensional Nanomaterials. Small.

[69] Pradhan B., Setyowati K., Liu H., Waldeck D.H., Chen J. (2008). Carbon Nanotube−Polymer Nanocomposite Infrared Sensor. Nano Lett..

[70] Zheng W., Li X., Dong C., Yan X., He G. (2014). Fabrication of a visible light detector based on a coaxial polypyrrole/TiO_2_ nanorod heterojunction. RSC Adv..

[71] Kotter D.K., Novack S.D., Slafer W.D., Pinhero P. (2008). Solar Nantenna Electromagnetic Collectors. Solar Thermal and Photovoltaic Power, Proceedings of the ASME 2008 2nd International Conference on Energy Sustainability, Jacksonville, FL, USA, 10–14 August 2008.

[72] Fang Z., Liu Z., Wang Y., Ajayan P.M., Nordlander P., Halas N. (2012). Graphene-Antenna Sandwich Photodetector. Nano Lett..

[73] Kumar S., Kumar S., Sengar M., Kumari P. (2021). Gold-carbonaceous materials based heterostructures for gas sensing applications. RSC Adv..

[74] Ryan T.J., Arnold K.J. (2011). Residential Carbon Monoxide Detector Failure Rates in the United States. Am. J. Public Health.

[75] Liu X., Cheng S., Liu H., Hu S., Zhang D., Ning H. (2012). A Survey on Gas Sensing Technology. Sensors.

[76] Zaporotskova I.V., Boroznina N.P., Parkhomenko Y.N., Kozhitov L.V. (2016). Carbon nanotubes: Sensor properties. A review. Mod. Electron. Mater..

[77] Akbari E., Buntat Z., Ahmad M.H., Enzevaee A., Yousof R., Iqbal S.M.Z., Ahmadi M.T., Sidik M.A.B., Karimi H. (2014). Analytical Calculation of Sensing Parameters on Carbon Nanotube Based Gas Sensors. Sensors.

[78] Paulla K.K., Farajian A.A. (2013). Concentration Effects of Carbon Oxides on Sensing by Graphene Nanoribbons: Ab Initio Modeling. J. Phys. Chem. C.

[79] Osborn T.H., Farajian A.A. (2014). Silicene nanoribbons as carbon monoxide nanosensors with molecular resolution. Nano Res..

[80] Olawoyin R. (2018). Nanotechnology: The future of fire safety. Saf. Sci..

[81] Wang Z., Wu S., Wang J., Yu A., Wei G. (2019). Carbon Nanofiber-Based Functional Nanomaterials for Sensor Applications. Nanomaterials.

[82] Li W., Zhang L.-S., Wang Q., Yu Y., Chen Z., Cao C.-Y., Song W.-G. (2012). Low-cost synthesis of graphitic carbon nanofibers as excellent room temperature sensors for explosive gases. J. Mater. Chem..

[83] Zhang J.T., Zhu Z.J., Chen C.M., Chen Z., Cai M.Q., Qu B.H., Wang T.H., Zhang M. (2018). ZnO-carbon nanofibers for stable, high response, and selective H2S sensors. Nanotechnology.

[84] Claramunt S., Monereo O., Boix M., Leghrib R., Prades J.D., Cornet A., Merino P., Merino C., Cirera A. (2013). Flexible gas sensor array with an embedded heater based on metal decorated carbon nanofibers. Sens. Actuators B Chem..

[85] Lee J.S., Kwon O.S., Shin D.H., Jang J. (2013). WO3 nanonodule-decorated hybrid carbon nanofibers for NO2 gas sensor application. J. Mater. Chem. A.

[86] Abideen Z.U., Kim J.-H., Lee J.-H., Kim J.-Y., Mirzaei A., Kim H.W., Kim S.S. (2017). Electrospun Metal Oxide Composite Nanofibers Gas Sensors: A Review. J. Korean Ceram. Soc..

[87] Katoch A., Choi S.-W., Sun G.-J., Kim H.W., Kim S.S. (2014). Mechanism and prominent enhancement of sensing ability to reducing gases in p/n core–shell nanofiber. Nanotechnology.

[88] Lee J.-H., Katoch A., Choi S.-W., Kim J.-H., Kim H.W., Kim S.S. (2015). Extraordinary Improvement of Gas-Sensing Performances in SnO2 Nanofibers Due to Creation of Local p–n Heterojunctions by Loading Reduced Graphene Oxide Nanosheets. ACS Appl. Mater. Interfaces.

[89] Li C., Feng C., Qu F., Liu J., Zhu L., Zhu Lin Y., Wang Y., Li F., Zhou J., Ruan S. (2015). Electrospun Nanofibers of p-Type NiO/n-Type ZnO Heterojunction with Different NiO Content and Its Influence on Trimethylamine Sensing Properties. Sens. Actuators B.

[90] Fink M., Harms R., Hatak I. (2012). Nanotechnology and Ethics: The Role of Regulation Versus Self-Commitment in Shaping Researchers’ Behavior. J. Bus. Ethics.

[91] Wang Z., Pan X., He Y., Hu Y., Gu H., Wang Y. (2015). Piezoelectric Nanowires in Energy Harvesting Applications. Adv. Mater. Sci. Eng..

[92] United States National Nanotechnology Initiative (US NNI). https://www.nano.gov/.

[93] Kogut D. (2019). Final Report for the Fire Extinguishing Performance Test of the Low Global Warming Potential (GWP) Agents. Report No ATC-12480. U.S. Army Aberdeen Test Center Aberdeen Proving Ground, MD 21005-5059; U.S. Army Ground Vehicle Systems Center (GVSC) Warren, MI 48397-5000. U.S. Army Aviation and Missile Command/Program Executive Office (PEO), Aviation Redstone Arsenal, AL 35898-5000. https://apps.dtic.mil/sti/pdfs/AD1079726.pdf.

[94] Łukaszczuk P. (2016). Zastosowanie nanotechnologii w ochronie przeciwpożarowej. Bezp. Tech. Pożar..

[95] Quintiere J.G. (2006). Fundamentals of Fire Phenomena.

[96] Han Z., Zhang Y., Du Z., Xu F., Li S., Zhang J. (2017). New-type gel dry-water extinguishants and its effectiveness. J. Clean. Prod..

[97] Martin T.J., Stevenson P. (2012). Fire-Fighting Foam Technology. Foam Engineering: Fundamentals and Applications.

[98] Du D., Shen X., Feng L., Hua M., Pan X. (2019). Efficiency characterization of fire extinguishing compound superfine powder containing Mg (OH). J. Loss Prev. Process. Ind..

[99] Leng N.B., Wang S.X., Han P. (2012). Development of New Fire Extinguishing Agent for Grassland. Adv. Mater. Res..

[100] Rueda-Núñez J.L. (2014). Chemical Composition for Fighting Forest Fires and Process for Obtaining Thereof. U.S. Patent.

[101] Han Y., Zhang X., Wu X., Lu C. (2015). Flame Retardant, Heat Insulating Cellulose Aerogels from Waste Cotton Fabrics by in Situ Formation of Magnesium Hydroxide Nanoparticles in Cellulose Gel Nanostructures. ACS Sustain. Chem. Eng..

[102] Vinogradov A.V., Kuprin D., Abduragimov I., Kuprin G., Serebriyakov E., Vinogradov V.V. (2016). Silica Foams for Fire Prevention and Firefighting. ACS Appl. Mater. Interfaces.

[103] Zhou R., Dou X., Lang X., He L., Liu J., Mu S. (2018). Foaming ability and stability of silica nanoparticle-based triple-phase foam for oil fire extinguishing: Experimental. Soft Mater..

[104] Zhao G., Xu G., Jin S., Zhang Q., Liu Z. (2019). Fire-Extinguishing Efficiency of Superfine Powders under Different Injection Pressures. Int. J. Chem. Eng..

[105] Hu W., Yu R., Chang Z., Tan Z., Liu X. (2021). The fire extinguishing mechanism of ultrafine composite dry powder agent containing Mg (OH). Int. J. Quantum Chem..

[106] Ivanovich Z.B. (2017). Method of Firefighting Using a Nano-Powder and Device for Its Implementation. Russian Patent.

[107] ABS (2017). Guidance notes on Fire-Fighting Systems. American Bureau of Shipping.

[108] Mosina K.S., Nazarova E.A., Vinogradov A.V., Vinogradov V.V., Krivoshapkina E.F., Krivoshapkin P.V. (2020). Alumina Nanoparticles for Firefighting and Fire Prevention. ACS Appl. Nano Mater..

[109] Cuffari B. How Nanotechnology is Improving Fire Safety Measures. AZONano, 2020. https://www.azonano.com/article.aspx?ArticleID=5485.

[110] Prashantha K., Lacrampe M.F., Krawczak P. (2011). Processing and characterization of halloysite nanotubes filled polypropylene nanocomposites based on a masterbatch route: Effect of halloysites treatment on structural and mechanical properties. Express Polym. Lett..

[111] Dubois P., Alexandre M., Claes M., Devalckenaere M. (2007). Fireproof Composition. Word Patent.

[112] Tran M.-P., Detrembleur C., Alexandre M., Jerome C., Thomassin J.-M. (2013). The influence of foam morphology of multi-walled carbon nanotubes/poly(methyl methacrylate) nanocomposites on electrical conductivity. Polymer.

[113] Xanthos M. (2010). Functional Fillers for Plastics.

[114] Leszczyńska A., Njuguna J., Pielichowski K., Banerjee J.R. (2007). Polymer/montmorillonite nanocomposites with improved thermal properties: Part I. Factors influencing thermal stability and mechanisms of thermal stability improvement. Thermochim. Acta.

[115] Kashiwagi T., Gilman J.W., Buter K.M., Harris R.H., Shields J.R., Asano A. (2000). Flame retardant mechanism of silica gel/silicas. Fire Mater..

[116] Hornsby P.R., Rothon R.N. (2005). Fire Retardant Fillers for Polymers in Fire Retardancy of Polymers: New Applications of Mineral Fillers.

[117] Salaün F., Lemort G., Butstraen C., Devaux E., Capon G. (2017). Influence of silica nanoparticles combined with zinc phosphinate on flame retardant properties of PET. Polym. Adv. Technol..

[118] Hamdani S., Longuet C., Perrin D., Lopez-Cuesta J.-M., Ganachaud F. (2009). Flame retardancy of silicone-based materials. Polym. Degrad. Stab..

[119] Osman M.A., Atallah A., Suter U.W. (2002). Reinforcement of poly(dimethylsiloxane) networks by montmorillonite platelets. J. Appl. Polym. Sci..

[120] Wypych G. (2018). Functional Fillers: Chemical Composition, Morphology, Performance, Applications.

[121] Gardelle B., Duquesne S., Rerat V., Bourbigot S. (2013). Thermal degradation and fire performance of intumescent silicone-based coatings. Polym. Adv. Technol..

[122] Gardelle B., Duquesne S., Vandereecken P., Bellayer S., Bourbigot S. (2013). Resistance to fire of intumescent silicone based coating: The role of organoclay. Prog. Org. Coat..

[123] Chimie R., Shin-Etsu Chemical Co., Ltd. (2014). Use of a Pretreated Precipitated Silica as a Reinforcing Filler for Silicon Elas-tomer and the Curable Silicone Elastomer Compositions thus Obtained by Cold Mixing. U.S. Patent.

[124] Nodera A., Kanai T. (2006). Flame retardancy of polycarbonate–polydimethylsiloxane block copolymer/silica nanocomposites. J. Appl. Polym. Sci..

[125] Maciejewski H., Dutkiewicz M., Byczynski L., Marciniec B. (2012). Silsesquioxanes as nanofillers. Part I. Silicone matrix nano-composites. Polimery.

[126] Cordes D., Lickiss P.D., Rataboul F. (2010). Recent Developments in the Chemistry of Cubic Polyhedral Oligosilsesquioxanes. Chem. Rev..

[127] Rezakazemi M., Vatani A., Mohammadi T. (2015). Synergistic interactions between POSS and fumed silica and their effect on the properties of crosslinked PDMS nanocomposite membranes. RSC Adv..

[128] Gilman J.W., Jackson C.L., Morgan A.B., Harris R., Manias E., Giannelis E.P., Wuthenow M., Hilton D., Phillips S.H. (2000). Flammability Properties of Polymer−Layered-Silicate Nanocomposites. Polypropylene and Polystyrene Nanocomposites. Chem. Mater..

[129] Hanu L., Simon G., Mansouri J., Burford R., Cheng Y. (2004). Development of polymer–ceramic composites for improved fire resistance. J. Mater. Process. Technol..

[130] Gilman J. (1999). Flammability and thermal stability studies of polymer layered-silicate (clay) nanocomposites. Appl. Clay Sci..

[131] Beall K.A. (1998). Annual conference on fire research. Annual Conference on Fire Research.

[132] Chang J.-H., Kim S.J., Joo Y.L., Im S. (2004). Poly(ethylene terephthalate) nanocomposites by in situ interlayer polymerization: The thermo-mechanical properties and morphology of the hybrid fibers. Polymer.

[133] Yano K., Usuki A., Okada A. (1997). Synthesis and properties of polyimide-clay hybrid films. J. Polym. Sci. Part A Polym. Chem..

[134] Blumstein A. (1965). Polymerization of adsorbed monolayers. II. Thermal degradation of the inserted polymer. J. Polym. Sci. Part A Gen. Pap..

[135] Costache M.C., Wang D., Heidecker M.J., Manias E., Wilkie C.A. (2006). The thermal degradation of poly (methyl methacrylate) nanocomposites with montmorillonite, layered double hydroxides and carbon nanotubes. Polym. Adv. Technol..

[136] Genovese A., Shanks R.A. (2008). Fire performance of poly(dimethyl siloxane) composites evaluated by cone calorimetry. Compos. Part A Appl. Sci. Manuf..

[137] Park E.-S. (2007). Mechanical properties and processibilty of glass-fiber-, wollastonite-, and fluoro-rubber-reinforced silicone rubber composites. J. Appl. Polym. Sci..

[138] (2000). Dow Silicones Corp. Flame Retardant Silicone Foams. U.S. Patent.

[139] Yasir M., Ahmad F., Yusoff P.S.M.M., Ullah S., Jimenez M. (2019). Latest trends for structural steel protection by using intumescent fire protective coatings: A review. Surf. Eng..

[140] Xu Z., Zhou H., Yan L., Jia H. (2019). Comparative study of the fire protection performance and thermal stability of intumescent fire-retardant coatings filled with three types of clay nano-fillers. Fire Mater..

[141] HasaneAhammad S., Rajesh V., Kumar K.S., Jalakam S., Kumar G.N.S. (2019). Statistical analysis of spinal cord injury severity detection on high dimensional MRI data. Int. J. Electr. Comput. Eng..

[142] Wang Z., Han E., Ke W. (2006). An investigation into fire protection and water resistance of intumescent nano-coatings. Surf. Coat. Technol..

[143] Lungu A., Cernencu A., Vlasceanu G., Florea N., Ionita M., Iovu H. (2021). 3D POSS cages decorated 2D graphenic sheets: A versatile platform for silicon-carbonaceous nano-additives design. Compos. Part B Eng..

[144] Rao T.N., Hussain I., Koo B.H. (2019). Enhanced thermal properties of silica nanoparticles and chitosan bio-based intumescent flame retardant Polyurethane coatings. Mater. Today Proc..

[145] Morys M., Illerhaus B., Sturm H., Schartel B. (2017). Variation of Intumescent Coatings Revealing Different Modes of Action for Good Protection Performance. Fire Technol..

[146] Akiike J. (2016). Slurry for Lithium Ion Secondary Battery Porous Film, Production Method Therefor, Separator for Lithium Ion Secondary Battery, and Lithium Ion Secondary Battery. U.S. Patent.

[147] Hefei Insulate New Mat Tech Co. Ltd (2017). Intumescent Fireproof Paint for Tunnel. CN Patent.

[148] Beaugendre A., Lemesle C., Bellayer S., Degoutin S., Duquesne S., Casetta M., Pierlot C., Jaime F., Kim T., Jimenez M. (2019). Flame retardant and weathering resistant self-layering epoxy-silicone coatings for plastics. Prog. Org. Coat..

[149] Kim Y., Lee S., Yoon H. (2021). Fire-Safe Polymer Composites: Flame-Retardant Effect of Nanofillers. Polymers.

[150] Laoutid F., Bonnaud L., Alexandre M., Lopez-Cuesta J.-M., Dubois P. (2009). New prospects in flame retardant polymer materials: From fundamentals to nanocomposites. Mater. Sci. Eng. R Rep..

[151] Sang B., Li Z.-W., Li X.-H., Yu L.-G., Zhang Z.-J. (2016). Graphene-based flame retardants: A review. J. Mater. Sci..

[152] Zielecka M., Rabajczyk A., Pastuszka Ł., Jurecki L. (2020). Flame Resistant Silicone-Containing Coating Materials. Coatings.

[153] Xu L., Wang Y.-Y., Huang J., Chen C.-Y., Wang Z.-X., Xie H. (2020). Silver nanoparticles: Synthesis, medical applications and biosafety. Theranostics.

[154] Sawicki K., Czajka M., Matysiak-Kucharek M., Fal B., Drop B., Męczyńska-Wielgosz S., Sikorska K., Kruszewski M., Kapka-Skrzypczak L. (2019). Toxicity of metallic nanoparticles in the central nervous system. Nanotechnol. Rev..

[155] Buccellato F.R., D’Anca M., Fenoglio C., Scarpini E., Galimberti D. (2021). Role of Oxidative Damage in Alzheimer’s Disease and Neurodegeneration: From Pathogenic Mechanisms to Biomarker Discovery. Antioxidants.

[156] Vinod C., Jena S. (2021). Nano-Neurotheranostics: Impact of Nanoparticles on Neural Dysfunctions and Strategies to Reduce Toxicity for Improved Efficacy. Front. Pharmacol..

[157] Bakand S., Hayes A. (2012). Finance Dechsakulthorn Nanoparticles: A review of particle toxicology following inhalation exposure. Inhal. Toxicol..

[158] Braakhuis H.M., Park M.V.D.Z., Gosens I., De Jong W.H., Cassee F.R. (2014). Physicochemical characteristics of nanomaterials that affect pulmonary inflammation. Part Fibre Toxicol..

[159] OSHA FactSheet Working Safely with Nanomaterials. https://www.osha.gov/sites/default/files/publications/OSHA_FS-3634.pdf.

[160] Technical ISO/TR, Report 18637 (2016). Nanotechnologies-Overview of Available Frameworks for the Development of Occu-Pational Exposure Limits and Bands for Nano-Objects and Their Aggregates and Agglomerates (NOAAs).

[161] NIOSH (2011). Occupational Exposure to Titanium Dioxide, Current Intelligence Bulletin 63.

[162] Rabajczyk A., Zielecka M. (2020). Emission of metal nanoparticles to the environment as a result of industrial processes. Chem. Ind..

[163] Wang J., Meng X., Yan K., Chen J. (2019). Suppression of Aluminum Dust Explosion by Ca(H_2_PO_4_)_2_/RM Composite Powder with Core–Shell Structure: Effect and Mechanism. Processes.

[164] Li G., Yang H.X., Yuan C.M., Eckhoff R.K. (2015). A catastrophic aluminum-alloy dust explosion in China. J. Loss Prev. Proc..

[165] Health and Safety Case Study: KunShan Explosion and Cancer at Samsung”. All Answers Ltd. ukdiss.com, November 2018. https://ukdiss.com/examples/health-and-safety-case-study.php?vref=1.

[166] Collin F. (2019). Chemical Basis of Reactive Oxygen Species Reactivity and Involvement in Neurodegenerative Diseases. Int. J. Mol. Sci..

[167] Nazbeen N., Wang Q. (2016). Guidelines and Best Practices for Safe Handling of Nanomaterials in Research Laboratories and Industries.

